# Lactylation in CNS disorders: mechanisms, cellular function, and disease relevance

**DOI:** 10.3389/fcell.2025.1566921

**Published:** 2025-03-28

**Authors:** Jiaxin Tian, Tongyu Zhang, Ruidan Zhang, Sijia Hao, Jingyu Dong, Yiyan Chen, Jinpeng Zhou, Yang Tian

**Affiliations:** ^1^ Department of Neurosurgery, Tangdu Hospital, Fourth Military Medical University, Xi’an, Shaanxi Province, China; ^2^ Department of Neurosurgery, Xuanwu Hospital, Capital Medical University, Beijing, China; ^3^ Department of Obstetrics, First Clinical College of Shanxi Medical University, Taiyuan, Shanxi Province, China

**Keywords:** lactylation, CNS disorders, mechanism, cellular function, disease pathology

## Abstract

Lactate, as a metabolic product or energy substrate, participates in various neurological processes within the physiological and pathological frameworks of the central nervous system (CNS). The groundbreaking application of multi-omics integration technologies has unveiled a novel role for lactate: lactylation, a unique post-translational modification (PTM) that covalently attaches lactate groups to lysine residues on proteins. This process precisely regulates protein function and gene expression, profoundly influencing the progression of various diseases. The lactylation process is meticulously regulated by a variety of key enzymes and metabolic pathways, forming a dynamic and intricate modification network. In this review, we summarize the key enzymes involved in lactylation, specifically “Writers,” “Erasers,” and “Readers.” Furthermore, we systematically categorize lactylation observed in various cell types within the CNS and investigate its multifaceted roles in pathological processes, including neurodegenerative diseases, brain tumors, and brain injuries. By consolidating the latest research findings in this field, our review aims to highlight the significance of these discoveries for future research and explore their potential for translational applications.

## 1 Introduction

**FIGURE 1:**
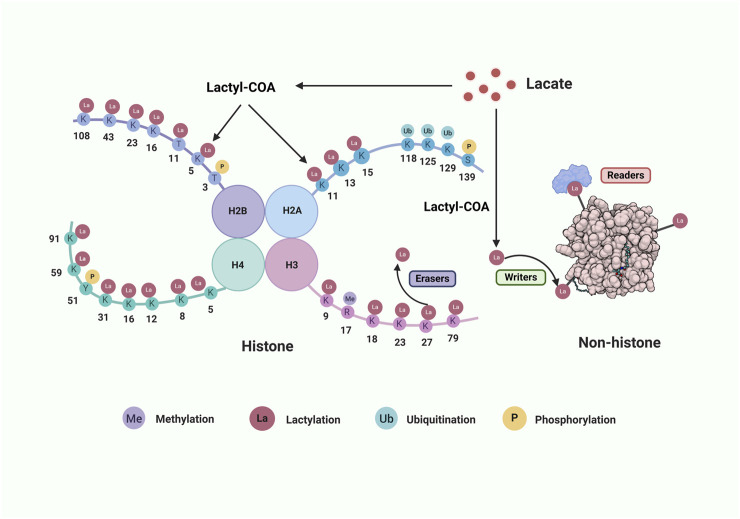
The lactylation process on histone and non-histone proteins. The core histone comprises H2B, H2A, H3 and H4. Lactate can be converted into lactyl-COA that serves as the substrate of lactylation on histone amino lysine acid residues (K) or non-histone. Many aminoacidic residues on histone tail are modified by various post-translational modifications. There also show three classes of crucial enzymes involved in protein lactylation. “Writers” are in green, “Erases” are in purple, and “Readers” are in red. Abbreviations: Me, methylation; La, lactylation; Ub, ubiquitination; P, phosphorylation. Created with BioRender.com.

Under hypoxic or low-oxygen conditions, lactate, a primary byproduct of glycolysis, plays a critical role in both physiological and pathological processes. It is worth mentioning that, even under aerobic conditions, tumor cells prefer to convert glucose into lactate to generate energy through glycolysis, and this special approach to produce lactate is also known as the Warburg effect (Liberti and Locasale, 2016; Sun et al., 2024a). Acting as both an energy substrate and a signaling molecule, lactate directly modulates cellular metabolism and function. Furthermore, it indirectly contributes to disease progression by shaping the local immune microenvironment, thereby highlighting its multifaceted roles in health and disease (Lee, 2021). Recent studies revealed that lactate could alter the structure of chromatin and gene transcription through epigenetic modification, leading to different biological effects (Visan, 2019). Lactylation is a new type of protein PTMs discovered by Professor Zhao of the University of Chicago in 2019 through mass spectrometry combined with liquid chromatography technology (Zhang et al., 2019). It has found that macrophages undergo lactylation modification of histone lysine residues under the influence of lactic acid, significantly regulating the transcriptional function of cells and affecting the phenotypic polarization of macrophages. Subsequently, scholars and scientists employed a variety of omics technologies to investigate lactylation, and successfully identified numerous lactylation modification sites (Figure 1) as well as its importance in development of diseases. As research progresses, lactylation in the condition of other diseases is gradually emerging. In the myocardial injury, renal ischemia-reperfusion, autoimmune diseases, and others, lactylation participates in the progression of physiological and pathological processes by influencing development, inflammation, energy metabolism, immune regulation, and other mechanisms. (Cuevas et al., 2024; Wang et al., 2022; Li et al., 2024d; Huang et al., 2024). Otherwise, during physical activity, the lactylation of two mitochondrial proteins PDHA1 and CPT2 under intracellular hypoxia halts oxidative phosphorylation (OXPHOS), which helps cells to respond quickly to low oxygen environments and prevents muscle damage caused by excessive exercise (Mao et al., 2024; Ren and Zhang, 2024; Wang et al., 2025).

The CNS is a highly active organ. Due to its characteristics of high oxygen consumption metabolism and low tolerance to hypoxia, lactate accumulation under pathological conditions catalyzes the occurrence of lactylation (Visan, 2019). In particular, the biological effects of lactylation are more significant in diseases such as neuro-oncology, ischemic and hypoxic encephalopathy, neurodegenerative disorders, and stroke. Therefore, this review systematically delineates the mechanisms and functions of lactylation in the CNS within the context of different diseases, aimed to provide possible research directions and valuable insights for related studies.

## 2 Overview of lactylation

Lactate serves not only as a metabolite of glycolysis but also as a critical energy substrate, playing a compensatory role in addressing adenosine triphosphate (ATP) shortages under hypoxic conditions when the tricarboxylic acid cycle is inhibited. Instead of entering the mitochondria for OXPHOS, pyruvate is directly converted into lactate through the catalytic activity of lactate dehydrogenase (Li et al., 2022b). Hypoxia, especially under pathological conditions, is frequently associated with elevated intracellular lactate levels. Beyond endogenous production, lactate can also be imported into cells through the activity of monocarboxylate transporters and sodium-coupled monocarboxylate transporters, further contributing to intracellular lactate accumulation (Fang et al., 2024). Numerous evidences have established that cellular metabolites serve as key substrates for protein PTMs. Notably, excess lactate predominantly drives the induction of a novel acylation modification termed lactylation.

Lactylation occurs through two distinct mechanisms: an enzymatic reaction that utilizes lactoyl-CoA as a substrate and a non-enzymatic reaction that employs lactoyl glutathione (LGSH) as a substrate (Gaffney et al., 2020; Xin et al., 2022). Enzymatic lactylation transfers the lactyl group from lactyl-CoA to lysine, catalyzed by lysine acetyltransferase and regulated by lactyl-CoA (Wu et al., 2023). Considering the non-enzymatic acyl-transfer mechanism, methylglyoxal, a highly reactive glycolytic byproduct, rapidly binds to glutathione through glyoxalase 1 to generate LGSH, which transfers its own lactyl group to a protein lysine residue (Gong et al., 2024; Sun et al., 2024b; Hu et al., 2024c; Gaffney et al., 2020). Histone lysine lactylation, alternatively named as Kla, has three isomers: Kl-la, N-ε-(carboxyethyl)-lysine (Kce), and d-lactyl-lysine (Kd-la). Among these, Kl-la is the predominant form and is dynamically regulated by glycolysis (Zhang et al., 2024a).

### 2.1 Techniques related to lactylation

With the evolution of sequencing technologies for genome-wide profiling of histone modifications and DNA-binding proteins, chromatin immunoprecipitation followed by sequencing (ChIP–seq) is the common method, but it has several limitations such as the requirement of a large number of input cells (Li et al., 2024c). Afterward, transposase-based single-cell techniques with improved signal-to-noise ratios were developed, such as CUT&Tag (Kaya-Okur et al., 2019), CoBATCH (Wang et al., 2019), scCUT&Tag-pro (Zhang et al., 2022), spatial-CUT&Tag (Deng et al., 2022) and Droplet Paired-Tag (Xie et al., 2023b). What’s more, the introduction of single-cell multimodal chromatin modification methods has facilitated simultaneous profiling of multiple histone modifications in the same cell, such as scMulti-CUT&Tag (Gopalan et al., 2021), MulTI-Tag (Meers et al., 2022), nano-CUT&Tag (nano-CT) (Bartosovic and Castelo-Branco, 2022), NTT–seq (Stuart et al., 2022) and uCoTarge (Xiong H. et al., 2024).

Nowadays, scholars tend to combine various innovative technologies to explore lactylation from different aspects. ATAC-seq, a technique used for analyzing chromatin accessibility, can be utilized in conjunction with CUT&Tag-seq to examine the effects of lactylation on chromatin accessibility, while RNA-seq can explore the influence of lactylation on gene expression through combing with these former two methods. However, these technologies have made great differences in uncovering lactylation, all these methods are based on next-generation sequencing platforms, which limit their applications to complex genomic regions, especially for repetitive elements accounting for 52% of human genome (Li et al., 2024c; Hoyt et al., 2022). Thus, improving and innovating current technologies are also quite important for supporting us to explore the unknown world of human genome secrets.

### 2.2 “Writers,” “erasers” and “readers” of lactylation

The process of protein lactylation is precisely regulated by a myriad of specialized enzymes which primarily consist of lactyltransferases that are responsible for adding lactyl-CoA and delactylases that are accountable for its removal. Furthermore, there are enzymes in charge of transforming lactate into lactyl-CoA, albeit they remain yet unidentified in mammals (Izzo and Wellen, 2019). The lactylation modification process is also reversible and dynamic, and these relevant enzymes are considered to represent the epigenetic activities of the “writer,” “eraser” and “readers” (Lin and Caroll, 2018; Li et al., 2023b; Xu et al., 2021; Chen et al., 2021; Yang et al., 2024b) (Figure 1).

#### 2.2.1 Writers

“Writers” are enzymes responsible for attaching acetyl groups to proteins. There are four major families of lysine lactyltransferases: p300/CREB-binding protein (p300/CBP) (Dancy and Cole, 2015; Zeng et al., 2023), MYST (HBO1, TIP60) (Yang et al., 2024b), GCN5-related N-acetyltransferase (GNAT) containing GCN5L2 and YiaC (Salah Ud-Din et al., 2016) as well as other lactyltransferase such as alanyl-tRNA synthetase 2 (AARS2) and alanyl-tRNA synthetase 1 (AARS1) (Li et al., 2024b; Zong et al., 2024; Ren and Zhang, 2024) (Table 1).

P300/CBP exhibits a significant and highly specific affinity for lactyl-CoA and is the first histone Kla writer to be identified (Hu et al., 2024c; Wang et al., 2024a; Xu et al., 2024). Extensive research has established that p300/CBP plays a pivotal role in orchestrating the lactylation of transcription factors, histones, and other nuclear proteins, thereby exerting far-reaching impacts on a diverse array of biological functions. For example, p300/CBP can regulate histone lactylation in macrophages (Yang et al., 2021; Zhang et al., 2019), as well as induced pluripotent stem cells (iPSCs) (Hu et al., 2024b; Li et al., 2022b), and serves as a “writer” of lactylation on the YTH N6-methyladenosine RNA-binding protein 2 (YTHDF2) promoter in ocular melanoma cells (Yu et al., 2021).

In MYST family, HBO1 is a classic lactyltransferase that mediates a Kla-dependent gene transcription and preferentially catalyzes H3K9 lactylation which significantly promotes tumorigenesis (Niu et al., 2024). TIP60, another important member of MYST family, is responsible for the lactylation of NBS1 at K388, which boosts homologous recombination-mediated DNA repair and enhances patients’ resistance to chemotherapy (Chen et al., 2024). TIP60 mediates vacuolar protein sorting 34 lipid kinase lactylation at lysine-356 and lysine-781 that promotes autophagic flux and endolysosomal trafficking (Jia et al., 2023).

It is particularly noteworthy that some enzymes may exhibit different catalytic roles during the modification process. A study revealed that HDAC6, traditionally considered a delactylase enzyme, can act as a primary lactyltransferase catalyzing the lactylation of α-tubulin. This modification enhances microtubule dynamics and promotes neurite outgrowth and branching in cultured hippocampal neurons (Sun et al., 2024b). While several lactyltransferases have been identified, a detailed understanding of the precise molecular mechanisms through which these enzymes function as “writers” remains lacking.

#### 2.2.2 Erasers

According to current studies, deacetylases can be broadly classified into two categories: histone deacetylases (HDACs) and other enzymes (Table 1). HDACs can be further divided into four classes: Class I–IV. Based on their cofactors, they are categorized as Zn^2+^-dependent enzymes (Class I, II, and IV, composed of HDAC1–11) and NAD^+^-dependent enzymes (Class III, composed of Sirtuin proteins 1–7) (Yang et al., 2024b; Moreno-Yruela et al., 2022). Specifically, Class I enzymes (HDAC1–3, 8) are primarily reported as delactylases and localized in the nucleus, while Class II enzymes (HDAC4–7, 9–10) and Class IV enzymes (HDAC11) typically shuttle lactyl-CoA between the nucleus and cytoplasm (Xu et al., 2021). Interestingly, certain members of the HDAC family exhibit multifunctionality, acting as both delactylases and deacetylases in various cellular contexts.

Continued research on NAD-dependent enzymes, particularly the extensively studied Sirtuin proteins (SIRT1–3), has deepened our understanding of delactylases. SIRT1, for instance, removes lactylation from α-myosin heavy chain, thereby protecting myocardial structure and function from damage in heart failure (Zhang et al., 2023c). SIRT2 serves as an efficient “eraser” of multiple histone lactylation sites, targeting synthetic histone peptides, purified histones, nucleosomes, and histones in neuroblastoma (NB) cells (Zu et al., 2022). SIRT3 demonstrates higher affinity for removing modifications involving hydrophilic hydroxyl motifs, facilitated by its unique hydrophobic pocket, which specifically accommodates the hydrocarbon portion of lactyl-lysine. This unique structural feature influences its binding mechanism and enhances its deacetylation activity (Xu et al., 2024). Nevertheless, the delactylation capacities of other SIRT family members remain poorly understood, highlighting the need for further in-depth investigation.

#### 2.2.3 Readers

“Readers” are specialized proteins capable of recognizing and transmitting lactylation signals by binding to lactate-modified proteins. Recent studies have identified bromodomain-containing proteins as potential lactylation readers, with the ability to recognize lactylation modifications and regulate the transcriptional activity of target genes (Cochran et al., 2019). Bromodomains, which are protein modules consisting of approximately 110 amino acids, specifically recognize lactylated lysine residues in both histone and non-histone proteins. Key examples of bromodomain-containing proteins include histone acetyltransferases, such as CREB-binding protein (CREBBP), p300, p300/CBP-associated factor (PCAF), and general control nonderepressible 5 (GCN5) (Cochran et al., 2019; Humphreys et al., 2017). In 2024, Hu et al. demonstrated that Brg1, a critical chromatin remodeling protein, acts as a histone lactylation reader. Brg1 specifically binds to H3K18la and accumulates at the promoters of pluripotency and epithelial junction-related genes, thereby enhancing their transcription, promoting cell phenotypic transitions, and improving the reprogramming efficiency of induced iPSCs (Hu et al., 2024b). Additionally, plant homeodomain finger protein 14 (PHF14), which contains a unique PHD1/Znk/PHD2 domain, has been identified as a novel lactylation reader. PHF14 has been shown to recognize the flexible N-terminal tail of histone H3 (amino acids 1–34) and specific PTM marks (Zheng et al., 2021). Despite these advancements, lactylation-specific readers remain largely unexplored, and the precise molecular mechanisms through which they transmit lactylation signals are still unclear. This knowledge gap likely reflects the novelty of lactylation as a PTM and its relatively recent discovery. A comprehensive investigation into the mechanistic intricacies of lactylation “readers” and their functional roles in cellular processes holds profound significance for the development of targeted therapeutic strategies aimed at modulating lactylation sites. Such efforts are anticipated to pave the way for advancements in clinical translation and the implementation of precision medicine approaches (Jing et al., 2024) (Table 1).

**TABLE 1:** Critical enzymes regulating the process of lactylation.

Function	Classification	Protein	Organism	Amino acids	References
Writer	P300/CBP family	P300	Mammals	2,414	Zhang et al. (2019), Dong et al. (2024), Yang et al. (2021), Hu et al. (2024c)
		CBP	Mammals	2,441	Yang et al. (2021), Hu et al. (2024c)
	MYST family	TIP60 (KAT5)	Mammals	513	Jia et al. (2023)
		HMOF (KAT8)	Mammals	467	Xie et al. (2024), Zou et al. (2024)
		HBO1 (KAT7)	Mammals	611	Niu et al. (2024)
	GNAT family	KAT2A^a^	Mammals	837	Wang et al. (2024d), Wang et al. (2022), Zhu et al. (2025)
		YiaC	*Escherichia coli*	231	Dong et al. (2022)
	Others	AARS2	Mammals	985	Ren and Zhang (2024), Mao et al. (2024), Li et al. (2024b)
		AARS1	Mammals	968	Ren and Zhang (2024), Li et al. (2024b), Zong et al. (2024), Ju et al. (2024)
		HDAC6	Mammals	various isomers	Sun et al. (2024b)
		HDAC3	Mammals	428	Sun et al. (2024b)
Eraser	Sirtuins (SIRTs) (Zn^2+^- dependent deacetylases)	SIRT1	Mammals	747	Zu et al. (2022)
		SIRT2	Mammals	489	Zu et al. (2022), Sun et al. (2024b)
		SIRT3	Mammals	187	Zu et al. (2022), Jin et al. (2023), Mao et al. (2024)
		SIRT5	Mammals	four isomers (301, 285, 262, 193)	Zu et al. (2022)
	Class I histone deacetylases (HDACs) (NAD^+^- dependent deacetylases)	HDAC1‒3	Mammals	HDAC1 —— 482 HDAC2 —— 488 HDAC3 —— 428	Moreno-Yruela et al. (2022)
	Others	CobB	*Escherichia coli*	231	Dong et al. (2022)
Reader	Unclear	Brg1	Mammals	1,647	Hu et al. (2024b)
		PHF14	Mammals	888	Zheng et al. (2021)

^a^ The common name of KAT2A is GCN5, hGCN5, GCN5L2, PCAF-b.

## 3 Lactylation in central nervous system

**FIGURE 2:**
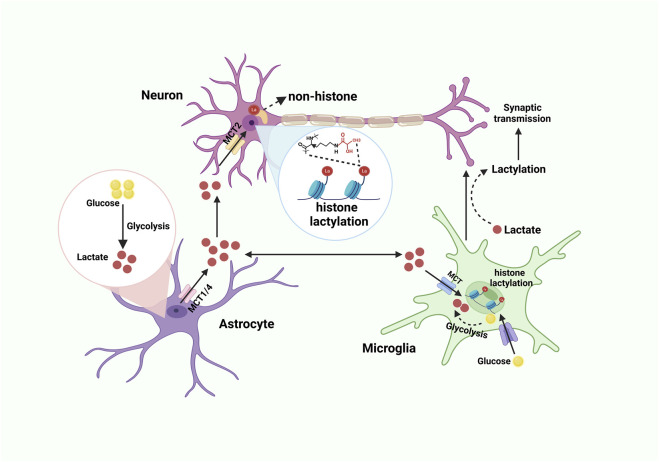
Lactate shuttle and lactylation in the CNS. Glucose is converted into lactate through glycolysis. Lactate derived from astrocytes is transported to the extracellular matrix via lactate transporters MCT1 and MCT4. Neurons and microglia then take up this lactate through MCT. Within neurons, lactate serves as the substrate for both histone and non-histone lactylation. Meanwhile, glucose enters microglia through the glucose transporter (GLUT) and is converted into lactate via glycolysis, which can subsequently influence synaptic transmission. Created with BioRender.com.

Despite comprising merely 2% of the total body mass, the cerebral organ demands roughly 20% of the systemic oxygen and energy resources. This substantial requirement is largely allocated to the sustenance of neuronal membrane potential gradients, the facilitation of synaptic signaling processes, and the synthesis as well as the exocytosis of neurotransmitters. Apart from glucose, the human brain possesses the capability to utilize lactate as an alternative energetic substrate to fulfill its energy requirements under hypoxic circumstances. The astrocyte-neuron lactate shuttle (ANLS) demonstrates that lactate released by astrocytes can be taken up and metabolized by neurons involved in glutamatergic signaling pathways (Brooks, 2018; Gong et al., 2024) (Figure 2). Nowadays, lactate-induced lactylation has been discovered mainly present in the cerebral cortex and hippocampus, and immunoreactivity for Kla is located in glutamatergic neurons, γ-amino butyric acidergic neurons, astrocytes, and microglia (Li et al., 2023b). However, studies concentrating on lactylation in other CNS cells encompassing oligodendrocytes and ependymal cells remain relatively scant.

### 3.1 Lactylation in neurons

As proposed by the ANLS hypothesis, neurons stimulate astrocytes to convert greater amounts of glucose into lactate through glutamate release, with the resulting lactate subsequently serving as an energy source for adjacent neurons (Bélanger et al., 2011). However, the increase in lactate further promotes the occurrence of lactylation modifications (Li et al., 2023b) (Figure 2).

In 2021, Hagihara et al. discovered that stress-associated neural excitation can stimulate histone H1 lactylation, with Kla signals detected throughout the cells, including the cytoplasm, nucleus, and neurites in primary cultured neurons. Notably, increased histone H1 lactylation was found to be associated with decreased social behavior (Hagihara et al., 2021). Furthermore, the lactylation of cortical synaptic proteins, particularly those initiated by elevated lactate levels during persistent exercise, serves to augment cortical neural network activity and alleviates anxiety-like behaviors in mice enduring chronic stress. Among these, the lactylation of synaptosome-associated protein 91 emerges as crucial for preventing anxiety-like behaviors in mice subjected to chronic restraint stress (Yan et al., 2024). Trough genome-wide dynamic analysis of H3K18la, combined with ATAC-seq and RNA sequencing, researchers have identified a strong correlation between H3K18la and chromatin state as well as gene expression during neural development. Specifically, in the embryonic cortex, H3K18la plays a pivotal role in regulating neuronal differentiation and proliferation (Dai et al., 2022a).

Remarkably, aberrant lactylation in neurons can impair lactate utilization, subsequently affecting neuronal energy metabolism and resulting in neuronal dysfunction. Furthermore, it affects learning and memory functions by disrupting neurotransmitter transmission and impact neuroplasticity. Lactylation is involved in the occurrence and development of neurodegenerative diseases (Yang et al., 2024a). In Alzheimer’s disease (AD), differences of H4K12la intensity are obvious in neurons between 5xFAD and WT mice through immunofluorescence co-staining (Pan et al., 2022). Additionally, higher serum lactate exists in cerebral ischemic reperfusion injury (CIRI), and the inhibition of endogenous lactate decreased protein pan-lactylation and increased the infarct volume. Moreover, the downregulated lactylation level of HDAC6 in neurons exacerbated ischemic neuronal injury through disturbing calcium homeostasis with binding BiP (Binding immunoglobulin protein), an endoplasmic reticulum-related protein (Cao et al., 2022). However, the increased levels of protein lactylation in neurons can be suppressed by MCT2 inhibition (Wu et al., 2024b). Mechanistically, inhibition of MCT2 results in decreased neuronal uptake of lactate and attenuates the lactate-induced expression of synaptic proteins post-synaptic density 95 (PSD95), synaptophysin (SYP), and growth associated protein 43 (GAP43), which are vital for synaptic transmission and significantly contribute to memory formation and retention (Wu et al., 2024b). Essentially, MCT2 antagonists can exacerbate the memory loss in neurodegenerative disorders. Along with other downregulators such as P300, these marks may become therapeutic targets to clinically protect neuronal functions.

### 3.2 Lactylation in astrocyte

Astrocytes originate from common neural progenitor cells with neurons (Wang et al., 2024c), and they are generally believed to be the primarily supporters of neurons. Serving as the primary glycogen storage in CNS, astrocytes are responsible for the production and release of lactate, which is transported to neurons via the ANLS pathway (Figure 2). Even in a resting state, lactate concentrations within astrocytes are much higher than within neurons (Kasparov, 2016).

The lactylation process within astrocytes plays a critical role in regulating neuronal functions (Cai et al., 2024), including memory consolidation and the modulation of vigilance and arousal effects on memory performance (Santello et al., 2019). In an animal model of cerebral ischemia-reperfusion, astrocytic low-density lipoprotein receptor-related protein-1 (LRP1) promotes the transfer of healthy mitochondria from astrocytes to neurons by reducing lactate production and inhibiting the lactylation of ADP-ribosylation factor 1 (ARF1), thereby enhancing neuronal recovery and alleviating neuropathological damage (Zhou et al., 2024).

However, aberrant lactylation can disrupt lactate synthesis or transport mechanisms, thereby impairing neurotransmitter signaling pathways and potentially contributing to neurological dysfunction. Specifically, the absence of the transcription factor BACH1 reduces lactate-dependent histone lactylation at the Lrrc15 promoter in microglia, which subsequently affects astrogenesis via the CD248-JAK-STAT signaling pathway, ultimately leading to abnormal neuronal differentiation and the development of anxiety-like behaviors (Wang et al., 2024c). Thus, elucidating the intricate interplay between lactylation-associated signals derived from microglia, neurons, and other CNS cells, and the receptors expressed on astrocyte progenitors that govern astrogenesis during embryonic development, represents a profound and multifaceted challenge in the field.

### 3.3 Lactylation in microglia

Microglia, the resident immune cells of the CNS, exhibit substantial heterogeneity and are indispensable for the regulation of immune responses and the maintenance of CNS homeostasis (Kabba et al., 2017). Under pathological conditions, microglia undergo metabolic reprogramming, transitioning from OXPHOS to aerobic glycolysis, resulting in the production of significant amounts of lactate. This lactate subsequently drives the secretion of pro-inflammatory cytokines, including TNF-α, IL-6, and IL-1β, which further exacerbate tissue damage and inflammation (Yang et al., 2024a) (Figure 2).

Beyond its role in metabolism, lactate within microglia also exerts regulatory effects on gene transcription through epigenetic modifications. Han et al. demonstrated that the accumulation of lactate resulting from exercise drives the transition of microglia from a pro-inflammatory to an anti-inflammatory phenotype via histone lactylation, thereby alleviating cognitive dysfunction (Han et al., 2023). In glioblastoma (GBM), lactylation regulation in microglia results in a decreased expression of homeostatic transforming growth factor-β (TGF-β) and impaired sensory functions, while concurrently upregulating genes associated with phagocytic activity (Monsorno et al., 2022; Maas et al., 2020). In the context of autoimmune uveitis, Yin-Yang 1 (YY1) lactylation significantly enhances the proliferative and migratory capacities of microglia (Huang et al., 2024). Moreover, YY1 lactylation in microglia transcriptionally activates and upregulates the expression of the key angiogenic factor FGF2, thereby facilitating retinal neovascularization and angiogenesis (Wang et al., 2023a; Chai et al., 2024). Moreover, microglia contribute to the pathogenesis of AD through the glycolysis/H4K12la/PKM2 (pyruvate kinase M2) positive feedback loop (Zhao and Xu, 2022), highlighting this pathway as a potential target for pharmacological intervention.

## 4 Lactylation in CNS disorders

### 4.1 Lactylation in CNS tumors

Tumor cells preferentially generate ATP through aerobic glycolysis rather than OXPHOS, even in the presence of sufficient oxygen. This metabolic reprogramming, commonly referred to as the “Warburg effect” (Wang et al., 2023b), is recognized as a defining hallmark of tumors (Yang et al., 2014). The Warburg effect has a critical impact on diverse cellular activities that transcend its metabolic effects, and it potentially supports a metabolic environment that allows for the rapid biosynthesis to support the uncontrolled growth and proliferation of tumor cells (Liberti and Locasale, 2016). Additionally, it is associated with increased DNA damage repair, and intervention in this regulatory process is promisingly considered as a potential way of sensitizing GBM to DNA damage-inducing therapies (Sun et al., 2024a).

Distinct from its role in healthy tissues, histone Kla has emerged as a crucial of oncogene expression and a key driver of tumor progression. Kla is intricately involved in various processes within the tumor microenvironment, including the orchestration of immune state transitions and the enhancement of tumor malignancy (Yang et al., 2024b). Furthermore, lactylation plays a crucial role in regulating glycolysis and macrophage polarization in tumor cells, thereby influencing tumor growth and immune suppression through the modulation of gene expression. As a result, targeting the glycolytic switch marked by lactylation presents a novel and promising therapeutic strategy for combating tumors (Dai et al., 2022b).

**FIGURE 3:**
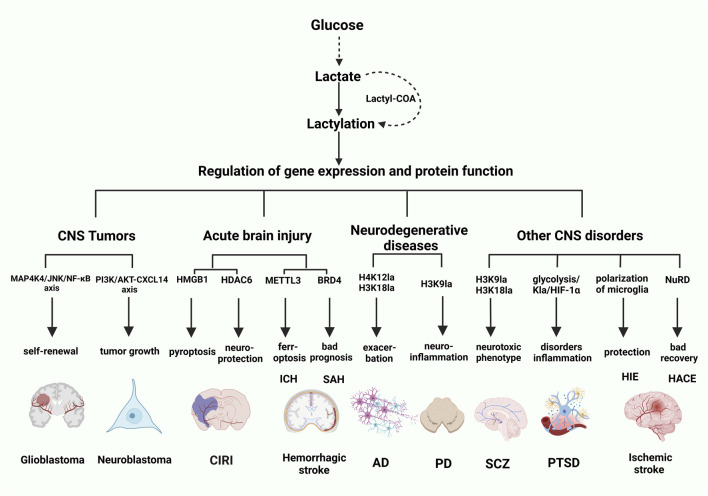
The diagram of the role and mechanism of lactylation in CNS disorders. Through the process of glycolysis, glucose is converted into lactate, which subsequently produces lactyl-CoA, serving as the substrate for lactylation. This modification plays an important role in a spectrum of CNS disorders through regulating gene expression and affecting protein functions. The various CNS disorders can be categorized into CNS tumors, acute brain injuries, neurodegenerative diseases, as well as other conditions such as SCZ, PTSD and ischemic stroke. Created with BioRender.com.

GBM, the most common malignant tumor of the CNS, predominantly relies on postoperative chemoradiotherapy to prolong patient survival. However, patients with high ALDH1A3-expressing GBM ALDH1A3hi GBM exhibit limited therapeutic benefits from such interventions. This limitation is primarily attributed to the interaction between ALDH1A3 and PKM2, which promotes the tetramerization of PKM2 and drives lactate accumulation and subsequent lactylation in GBM stem cells. Through proteomic analysis of lactylated proteins, Li et al. demonstrated that lactylation of *XRCC1* at lysine 247 (K247) enhances its binding affinity for importinα, thereby promoting increased nuclear translocation of *XRCC1* and facilitating DNA repair in tumor cells. Notably, disrupting the ALDH1A3-PKM2 interaction has been shown to potentiate chemoradiotherapy-induced apoptosis in GBM cells, offering a potential therapeutic strategy for ALDH1A3hi GBM (Li et al., 2024a). Glucose-driven histone lactylation has been shown to enhance the immunosuppressive activity of monocyte-derived macrophages (MDMs) (De Leo et al., 2024). Notably, the deletion of protein kinase R-like endoplasmic reticulum kinase in MDMs suppresses histone lactylation, resulting in the accumulation of intra-tumoral T cells and a significant delay in tumor growth. When combined with immunotherapy, this strategy demonstrates the potential to effectively inhibit the progression of GBM (De Leo et al., 2024). What’s more, histone lactylation-mediated upregulation of *LINC01127* promotes the self-renewal of GBM stem cells via cis-regulating mitogen-activated protein kinase 4 (MAP4K4) to activate JNK pathway (Li et al., 2023a). *LINC01127* is a lncRNA linked to the NF-κB pathway, and its high expression correlates with poor prognosis in GBM patients. Specifically, the Warburg effect-induced histone lactylation stimulates the expression of NF-κB-associated *LINC01127*, that subsequently fosters GBM cell self-renewal via the MAP4K4/JNK/NF-κB signaling axis (Li et al., 2023a) (Figure 3). Currently, CAR-T cell therapy is being gradually tested for the treatment of GBM (Maalej et al., 2023). Sun et al. found that intracellular lactate-induced H3K18la directly enhances the activity of the promoters for *CD39*, *CD73*, and *CCR8* genes. Inhibiting lactate production can alleviate immunosuppression within the tumor microenvironment and reduce tumor-infiltrating CAR-Treg cells, presenting a potential strategy to enhance the efficacy of CAR-T cell therapy (Sun et al., 2023).

NB, the most prevalent and lethal extracranial solid tumor in juveniles, is profoundly influenced by the dynamic interplay between tumor cells and tumor-associated macrophages, which play a critical role in driving tumor progression (Wu et al., 2024a). Emerging evidence indicates that elevated histone lactylation significantly enhances the proliferation and migration of NB cells, while SIRT2, acting as an “eraser” of histone lactylation, exhibits tumor-suppressive effects by mitigating NB progression (Zu et al., 2022). Furthermore, lactate secretion and HK3-mediated histone lactylation within NB cells have been shown to facilitate the recruitment and polarization of M2-like macrophages via the PI3K/AKT-CXCL14 axis (Figure 3), thereby contributing to the establishment of a tumor-promoting microenvironment and further accelerating NB progression (Wu et al., 2024a) (Table 2).

### 4.2 Lactylation in acute brain injury

Acute brain injury (ABI) is a complex and multi-faceted disorder characterized by severe damage to brain tissue or functional impairments, often resulting from sudden strokes, penetrating injuries, severe vehicular accidents, or combat-related trauma. In ABI patients, lactate plays a vital role in supplying energy to the brain (Brooks, 2018), and lactylation, triggered by lactate, is crucial in both the progression and recovery of the condition.

During the progression of CIRI, hypoxia-induced cellular stress significantly upregulates LDHA expression and activates the glycolytic pathway, resulting in elevated lactate production that serves as a critical substrate for protein lactylation. The accumulation of lactate during CIRI has been shown to stimulate the release of pro-inflammatory cytokines, including TNF-α, IL-6, and IL-1, thereby amplifying neuronal damage (Li et al., 2022b; Andersson et al., 2005). In 2023, Yao et al. conducted a comprehensive investigation of protein lactylation in brain endothelial cells of CIRI rats, identifying 54 upregulated and 54 downregulated lactylation sites. Their findings demonstrated that increased lactylation is closely linked to key processes such as energy metabolism, neuronal damage, repair mechanisms, and neurodevelopment (Yang et al., 2025; Yao et al., 2023). Furthermore, histone lactylation was specifically enriched in the promoter region of the high mobility group box 1 (HMGB1) gene. This enrichment upregulated HMGB1 expression, which in turn activated the nucleotide-binding oligomerization domain-like receptor protein 3 inflammasome, leading to caspase-1 activation, pyroptosis, and exacerbation of CIRI pathology (Yao and Li, 2023).

Lactylation has been shown to play a pivotal role in the pathophysiology of hemorrhagic stroke. Recent studies have demonstrated that in an intracerebral hemorrhage (ICH) model, the lactylation of methyltransferase-like protein 3 (METTL3) (Figure 3) was significantly upregulated, leading to enhanced stability and increased expression of the METTL3 protein. This upregulation further elevated the m6A modification and mRNA expression of the transferrin receptor, thereby promoting ferroptosis and contributing to the progression of ICH (Zhang et al., 2023a). These findings suggest that lactylation indirectly drives the pathogenesis of ICH through its regulatory impact on ferroptosis. Conversely, in a subarachnoid hemorrhage (SAH) model, histone lactylation was found to exert neuroprotective effects by inhibiting the polarization of the astrocyte A1 phenotype, thereby mitigating neuroinflammation and reducing neuronal death after SAH. Bromodomain-containing protein 4 (BRD4) (Figure 3) in astrocytes was identified as a critical regulator of histone lactylation in this process. Silencing of BRD4 markedly reduced H4K8la lactylation, which subsequently exacerbated the polarization of the astrocyte A1 phenotype, leading to impaired neuroprotection and worsening neurological outcomes (Zhang et al., 2024b). These findings highlight the dual and context-dependent roles of lactylation in hemorrhagic stroke (Table 2).

### 4.3 Lactylation in neurodegenerative disease

Disrupted glucose metabolism is a hallmark of AD (Yang et al., 2024a; Xiang et al., 2021; Liguori et al., 2016). Notably, lactate concentrations in the cerebrospinal fluid (CSF) of AD patients are significantly elevated compared to healthy individuals (Wang et al., 2024b). Pan et al. observed a marked increase in lactylation levels, particularly at the H4K12 site (Figure 3), in the brain tissues of 5xFAD mouse models and AD patients, with these levels correlating with the accumulation of Aβ plaques. A positive feedback loop involving glycolysis, H4K12la, and PKM2 has been identified as a key driver of microglial activation and dysfunction by upregulating the transcription of glycolytic genes. This mechanism exacerbates pathological processes, including Aβ deposition, tau hyperphosphorylation, synaptic damage, and cognitive decline, thereby contributing to the progression of AD (Pan et al., 2022; Zhao and Xu, 2022).

Recently, a study employed datasets GSE85426 and GSE97760 to identify candidate genes by intersecting weighted gene co-expression network analysis module genes with AD-control differentially expressed genes, which eventually uncovered three histone lactylation-linked genes (*ARID5B*, *SESN1*, *XPA*) as potential AD biomarkers. *SESN1* and *XPA* may influence AD development by affecting cellular senescence and cell cycle pathways. Additionally, *XPA* could improve β-amyloid precursor protein and tau pathology via the lysosomal pathway (Guo et al., 2024). The K677 mutation in tau protein may protect against AD by influencing ferritinophagy and ferroptosis via the MAPK signaling pathways. Mechanistically, the conversion of the K677 site to arginine (R) diminishes tau lactylation and suppresses ferroptosis by regulating iron metabolism factors like NCOA4 and FTH1 which improve memory and learning skills (An et al., 2024). Understanding the role of lactylation in tau protein could pave the way for innovative therapeutic strategies for AD.

The defining pathological feature of Parkinson’s disease (PD) is the progressive and selective degeneration of dopaminergic neurons in the substantia nigra and striatum. Recent studies have reported abnormally elevated lactate levels in the CSF of patients with late-onset PD (Yang et al., 2024a; Wang et al., 2024b). For example, the increased lactate production in PD promoted by the upregulated expression of hexokinase 2 can induce the apoptosis of dopaminergic neurons (Li et al., 2022a). Elevated lactate levels are hypothesized to enhance H3K9la modification at the promoter region of solute carrier family 7 member 11 (*SLC7A11*) (Figure 3), thereby upregulating its transcription. This upregulation contributes to microglial activation and neuroinflammatory damage. Importantly, inhibiting this pathway has been shown to reduce dopaminergic neuronal apoptosis and suppress the inflammatory state of microglia (Wang et al., 2024b; Nohesara et al., 2024) (Table 2).

### 4.4 Lactylation in other CNS disorders

In schizophrenia (SCZ), neuronal cells exhibit an abnormally active metabolic state and elevated levels of the senescence-associated secretory phenotype (Sfera et al., 2024). The aryl hydrocarbon receptor, as a master regulator of cellular senescence, has been shown to negatively affect premature neuronal and glial aging as well as the excessive lactylation observed in SCZ (Sfera et al., 2024). In SCZ, senescent microglia with lactylation at the H3K18 histone site exhibit neurotoxic properties, contributing to the elimination of healthy synapses and neurons. This indicates that lactylated neurotoxic microglia may pose a greater threat to severe psychiatric disorders than previously recognized (Sfera et al., 2024). Elevated levels of lactylation have been observed in both *in vivo* and *in vitro* MK801-induced SCZ models. Treatment with the glycolysis inhibitor 2-deoxy-D-glucose (2-DG) effectively reduced lactate accumulation and the lactylation of H3K9 and H3K18 (Figure 3), subsequently improving behavioral abnormalities in mice (Nohesara et al., 2024; Xie et al., 2023a).

Clinical metabolomic studies have identified lactate as a pivotal factor in the onset and progression of post-traumatic stress disorder (PTSD). The lactylation of HIF-1α and the neuroprotective protein membrane-organizing extension spike protein has been implicated in premature cellular senescence, contributing to the manifestation of depression and anxiety symptoms in individuals with PTSD (Figure 3). This process is recognized as a primary factor underlying the pathogenesis of PTSD (Liu and Zhang, 2024; Kozlakidis et al., 2023; Sara et al., 2022). Furthermore, the maladaptive interplay between glycolysis, histone lactylation, and HIF-1α dysregulation exacerbates microglial glucose metabolism disturbances and inflammatory responses, thereby amplifying the clinical severity of PTSD (Liu and Zhang, 2024).

In neonatal hypoxic-ischemic encephalopathy (HIE), microglia are activated and secrete inflammatory cytokines that are harmful to neurons and glial cells, causing neuronal damage through the release of inflammatory factors. However, histone lactylation in HIE regulates the polarization state of microglia (Figure 3), shifting them from a pro-inflammatory phenotype to an anti-inflammatory phenotype, thereby promoting repair and healing effects (Zhou et al., 2022).

Through the application of advanced lactylation proteomics analysis, Jiang et al. comprehensively characterized the landscape of protein lactylation modifications in a validated high-altitude cerebral edema (HACE) mouse model. Their study identified a substantial induction of lysine-lactylated proteins under hypoxic conditions, which were predominantly enriched in critical protein complexes, including the NuRD complex (Figure 3), ribosomal biogenesis complex, spliceosome complex, and DNA replication complex. These lactylation modifications were found to exacerbate hypoxia-induced neuroinflammatory responses and significantly contribute to the pathological progression of HACE, providing novel insights into the molecular mechanisms underlying this condition (Jiang et al., 2024) (Table 2).

**TABLE 2:** Molecules susceptible to lactylation and its impacts in CNS disorders.

Diseases	Molecules susceptible to lactylation	Molecular function following lactylation	Key findings	References
AD	H3K18	Stimulate the NF-κB signaling pathway	H3K18la/NF-κB axis upregulates SASP components IL-6 and IL-8 by increasing binding to the promoter of Rela (p65) and NF-κB1 (p50) in AD	Wei et al. (2023)
	H4K12	Activate glycolytic genes’ transcription in microglia	Aglycolysis/H4K12la/PKM2 positive feedback loop exacerbates microglia dysfunction and drives the pathogenesis of AD	Pan et al. (2022)
	*ATG3*, *ATG5*, *ATG16L1*	Promote autophagy-related genes (*ATG3*, *ATG5*, *ATG16L1*) expression	The downregulated EPB41L4A-AS1 in AD inhibits Aβ clearance by regulating the expression of GCN5L2 affecting histone lactylation near the transcription start site of autophagy-related genes	Wang et al. (2024d)
PD	H3K9	Activate SLC7A11 expression	H3K9la is increased in the substantia nigra of PD mice that activates SLC7A11 and aggravates microglia-mediated neuroinflammation	Qin et al. (2025)
PTSD	HIF-1α	Promote endothelial cells’ premature aging	HIF-1α lactylation accelerates the premature aging of endothelial cells and triggers the onset of depression and anxiety in PTSD	Chen et al. (2022)
SCZ	H3K9, H3K18	Regulate gene or pathway activation	Alleviating lactylation prevents behavioural changes in mice with MK801-induced SCZ model probably in association with HMGB1	Xie et al. (2023a)
GBM	*XRCC1*	Improve DNA repair	Glycometabolic reprogramming-induced *XRCC*1 lactylation confers therapeutic resistance in ALDH1A3-overexpressing glioblastoma	Li et al. (2024a)
	*CCR8* H3K18	Elevate the activities of *CD39, CD73* and *CCR8* gene promotors	Oxamate enhances the efficacy of CAR-T therapy against GBM via suppressing *CCR8*lactylation	Sun et al. (2023)
	Histone	Promote IL-10 expression	PERK-driven glucose metabolism promotes the immunosuppressive activity of monocyte-derived macrophages in GBM via histone lactylation	De Leo et al. (2024)
	VEGFR2 VE-cadherin	Increase protein expression	The upregulated VEGFR2 and VE-cadherin lactylation in GBM cells promote its proliferation, migration, invasion, and VM development	Zhang et al. (2023b)
	H3K9	Activate *LUC7L2* transcription	H3K9la confers temozolomide resistance in GBM via *LUC7L2*-mediated intron 7 retention of MLH1	Yue et al. (2024)
	H3 histone	Activate NF-κB-related *LINC01127* expression	Histone lactylation-derived *LINC01127* promotes the self-renewal of GBM stem cells via the cis-regulating the MAP4K4 to activate JNK pathway	Li et al. (2023a)
SAH	H4K8	Aggravate the A1 polarization of astrocytes	The silenced BRD4 in astrocytes reduces H4K8la lactylation that aggravates the A1 polarization of astrocytes and affects the recovery of neural function and prognosis	Zhang et al. (2024b)
ICH	METTL3	Enhance METTL3 protein stability and expression levels	METTL3 silenced inhibites the ferroptosis development via regulating the TFRC levels in the ICH progression	Zhang et al. (2023a)
CIRI	HDAC6	Disturb calcium homeostasis	The downregulated lactylation level of HDAC6 in neurons exacerbates ischemic neuronal injury	Cao et al. (2022)
HACE	Protein complexes^a^	Regulate protein function and contributes to the neuro-inflammatory response	Lactylation in the NuRD complex changes its 3D structure that regulates protein function and contributes to the inflammatory response in microglia	Jiang et al. (2024)
Spinal cord injury (SCI)	H4K12	Promote PD-1 transcription in microglia and facilitate SCI repair	H4K12la promotes PD-1 transcription and enhances microglial scar formation and functional recovery after SCI	Hu et al. (2024a)
Ischemic stroke	Protein lysine	Exacerbate neurons’ death	protein Kla promoted by astrocytes-derived lactate aggravates ischemic brain injury	Xiong X. Y. et al. (2024)

^a^ Protein complexes mainly include NuRD complex, ribosome biogenesis complex, spliceosome complex, and DNA replication complex.

Abbreviations: SASP: senescence-associated secretory phenotype; *CCR8*:chemokine (C-Cmotif) receptor 8; IL-10: interleukin-10; PERK: ER kinase; VEGFR2:vascular endothelial growth factor receptor 2; VE-cadherin: vascular endothelial cadherin; VM: vasculogenic mimicry; TFRC: transferrin receptor.

## 5 Conclusion and perspectives

As a recently discovered PTM, lactylation remains in the nascent stages of research but has shown remarkable potential in regulating both physiological and pathological processes within the CNS. This modification exerts its regulatory effects on gene transcription, protein expression, and signal transduction primarily through modulating chromatin accessibility, facilitating the nuclear translocation of transcription factors, and maintaining protein stability. Unlike traditional PTMs, lactylation exhibits dynamic changes that are tightly linked to cellular nutritional status, oxygen availability, and energy demands, thus playing a more integral role in orchestrating CNS functions, including energy metabolism, immune regulation, and developmental homeostasis. Consequently, a comprehensive and systematic exploration of the multifaceted roles of lactylation may offer valuable insights into the intricate physiological and pathological mechanisms underlying CNS function.

Lactylation research is currently experiencing a surge of activity; however, several critical questions persist that necessitate further exploration. Primarily, the comprehensive elucidation of the enzymes responsible for lactylation modification remains incomplete, particularly in the context of non-histone lactylation, which may highly depend on the facilitation of specific enzymes. While HDACs and Sirts have been recognized as pivotal players in delactylation processes, their involvement in additional functions, such as deacetylation, necessitates further validation of their substrate specificity and underlying regulatory mechanisms. Secondly, the mechanism underlying the site-specificity of lactylation remains an enigma. This intricacy encompasses diverse factors, including protein structure, microenvironmental impacts, and interactions with other PTM. Elucidating this aspect is indispensable for future precise targeting of lactylation sites. Furthermore, the intricate coupling between lactylation and metabolic dynamics remains obscure. As an intermediate metabolite, the mechanism by which lactate concentrations are “sensed” and translated into lactylation modification signals remains an area of significant inquiry. Lastly, the dearth of targeted intervention strategies for lactylation in disease contexts poses a significant challenge. The current lack of specific small-molecule tools or gene-editing methodologies aimed at lactylation modifications severely hinders translational medical research in this domain. Addressing these gaps will be crucial for advancing our understanding and harnessing the potential of lactylation in therapeutic applications.

This review provides a comprehensive overview of the current researches on lactylation modification in the CNS, highlighting its diverse roles and underlying molecular mechanisms across various neural cell types and disease contexts. Given the inherent heterogeneity of diseases, cellular diversity, and the intricate crosstalk between different PTMs in epigenetics, extensive further investigation is essential to advance the clinical translation of lactylation-related discoveries. The integration of emerging technologies, including single-cell sequencing, spatial transcriptomics, and machine learning, with lactylation omics offers significant potential for the precise identification of lactylation sites, detailed characterization of their functional impacts, and targeted intervention of disease-specific lactylation events in distinct pathological conditions.
